# Defensive Symbiont Genotype Distributions Are Linked to Parasitoid Attack Networks

**DOI:** 10.1111/ele.70082

**Published:** 2025-02-18

**Authors:** Taoping Wu, Anoushka A. Rodrigues, Tom M. Fayle, Lee M. Henry

**Affiliations:** ^1^ School of Biological and Behavioural Sciences Queen Mary University of London London UK; ^2^ Biology Centre of the Czech Academy of Sciences Institute of Entomology Ceske Budejovice Czech Republic

**Keywords:** defensive symbiosis, facultative symbionts, *Hamiltonella defensa*, horizontal transmission, host‐parasitoid networks, insect ecology

## Abstract

Facultative symbionts are widespread in arthropods and can provide important services such as protection from natural enemies. Yet what shapes associations with defensive symbionts in nature remains unclear. Two hypotheses suggest that interactions with either antagonists or host plants explain the prevalence of symbionts through shared selective pressures or vectors of symbiont transmission. Here we investigate the factors determining similarities in the *Hamiltonella defensa* symbiosis shared amongst field‐collected aphid species. After accounting for host species relatedness, we find that *Hamiltonella's* genotype distribution aligns with sharing the same parasitoids, rather than host plants, highlighting parasitoids and hosts as key selective agents shaping the symbiosis across aphid species. Our data indicates parasitoid host specificity drives the prevalence of specific aphid‐*Hamiltonella* associations, suggesting defensive symbioses are maintained by the selective pressure imposed by dominant parasitoids and their aphid hosts. These findings underscore the importance of interactions with natural enemies in explaining patterns of defensive symbiosis in nature.

## Graphical abstract

Facultative symbionts are widespread in arthropods and can provide important services such as protection from natural enemies. Yet what shapes defensive symbiont associations in nature remains unclear. We show that the symbiont *Hamiltonella defensa's* genotype distribution strongly aligns with sharing the same parasitoids, rather than host plants, highlighting parasitoids as a key selective agent shaping defensive symbioses across host species.

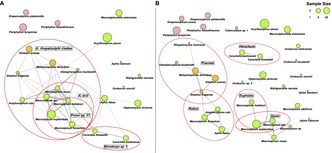

## 1 Introduction

Heritable bacterial symbionts are widespread in insects and often confer ecologically important traits to their hosts. Many insects harbour vertically transmitted obligate symbionts that have enabled expansions into novel feeding niches by provisioning their hosts with essential nutrients (Cornwallis et al. 2023; Jackson et al. 2022). Even more widespread are heritable facultative symbionts, which are not essential for hosts but can provide important context‐specific benefits, such as protection from biotic or abiotic stresses (Heyworth et al. 2020; McLean and Godfray 2015; Tougeron and Iltis 2022; Wu et al. 2022). Facultative symbionts can also be horizontally transferred between hosts (Henry et al. 2015; Russell et al. 2003) and tend to be non‐randomly distributed amongst plant‐adapted insect populations and species (e.g., Henry et al. 2015; Henry et al. 2022; Jackson et al. 2023; Toju and Fukatsu 2011; Wu et al. 2022), although the reasons for this are currently unknown. This has led researchers to propose that facultative symbionts may provide benefits to populations in certain ecological niches, with high frequencies of symbiont carriage due to the acquisition and spread of symbionts that confer local adaptations (Jaenike 2012). However, the role of facultative symbionts in insect ecology has been hampered by our limited understanding of symbiont exchange networks and the agents of selection shaping their populations.

Facultative symbionts have been shown to provide protection against natural enemies in several insect groups, including *Lagria* beetles, *Drosophila* flies, beewolves, whiteflies, and in aphids (e.g., Vorburger et al. 2010; Xie et al. 2014; Kaltenpoth et al. 2005; Flórez et al. 2017; Oliver and Perlman 2020; Zhao et al. 2023), and are likely undescribed in other systems as well. While laboratory studies have provided valuable insight into the biology of defensive symbioses, our understanding of their network ecology in the wild is limited. Defensive symbionts are often maintained at intermediate frequencies in host populations (e.g., Ferrari et al. 2012; Osaka et al. 2010; Henry et al. 2013, 2015; Zhao et al. 2023), suggesting a range of factors influence their spread and maintenance (Oliver et al. 2008; Smith et al. 2021). Selective forces such as the cost–benefit balance of maintaining symbionts in different ecological scenarios, as well as non‐selective factors such as transmission rates, migration, and drift, likely shape defensive symbiont distributions in host populations (reviewed in Vorburger 2022). Moreover, the horizontal transfer of facultative symbionts amongst host species complicates our understanding of how defensive symbioses function within insect communities, particularly those facing diverse natural enemies. Defensive symbiont function is therefore likely to have important implications for the dynamics of both natural ecosystems and agricultural landscapes, in which insects, such as aphids, are important pest groups (e.g., Leclair et al. 2021).

Some of the best‐known examples of defensive symbioses have emerged from the bacterial symbionts of aphids. In addition to their obligate nutritional symbiont, *Buchnera aphidicola* (Munson et al. 1991), aphids harbour an array of facultative symbiont species, several of which can confer protection against natural enemies (Ferrari et al. 2004; Oliver et al. 2003). Amongst these, *Hamiltonella defensa* (Moran et al. 2005), hereafter *Hamiltonella*, stands out for its widespread presence in aphid populations and its ability to prevent parasitoid development after attack. In nature, aphid species and plant‐adapted aphid ‘biotypes’, tend to be associated with only a few closely related *Hamiltonella* strains (Henry et al. 2013; Wu et al. 2022). However, the reason for this is currently unclear. *Hamiltonella* is strongly associated with aphids that feed on certain host plants, suggesting interactions with plants may shape its distribution (Gimmi et al. 2024; Henry et al. 2013, 2015). However, different strains of *Hamiltonella* can also protect against different species of parasitoid wasps (McLean and Godfray 2015). In nature, some aphids have retained *Hamiltonella* strains that provide strong protection against their main parasitoid species (Gimmi and Vorburger 2024; Wu et al. 2022), suggesting antagonists may contribute to the symbiont's distribution in nature. In addition, phylogenetic studies have shown that *Hamiltonella* can be transferred horizontally between unrelated aphid species (Henry et al. 2013; Wu et al. 2022), although how transfer occurs in nature is unclear. Ecological vectors may contribute to non‐random patterns of facultative symbiont infection by establishing a route of symbiont transmission between host species, even if incidences of horizontal transfer are rare in nature. Laboratory studies have shown that *Hamiltonella* can be transferred between hosts by parasitoids through a contaminated ovipositor (Gehrer and Vorburger 2012), but also via plant phloem (Li et al. 2018), suggesting that both mechanisms may contribute to the dynamics of symbiont acquisition and transmission in nature. However, the relative importance of plant and parasitoid interactions in shaping *Hamiltonella*'s presence in aphid communities remains unclear.

In this study, we analyse data from 1257 aphid samples to investigate ecological correlates linked to *Hamiltonella*'s genotype distribution. Specifically, we ask whether *Hamiltonella* strains found in different aphid species are best explained by sharing parasitoids or host plants to shed light on the ecological drivers of defensive symbiont dynamics in nature.

## 2 Materials and Methods

### 2.1 Aphid‐Parasitoid Sampling and Identification

In 2021 and 2022, live parasitised and ‘mummified’ (i.e., a parasitoid cocoon) aphids were collected from 17 plant species in 26 locations across Greater London, UK (Tables S1 and S2), focusing on aphid species known to harbour *Hamiltonella* (from Henry et al. 2015 and Wu et al. 2022). Aphids were collected by beating plants over a tray or manual removal from leaves and stems. To avoid resampling the same aphid clone or mummies from the same parasitoid, collections from the same plant species were spaced at least 10 m apart. Additionally, we included 241 *Hamiltonella* samples from unparasitized aphids from a previous study (Wu et al. 2022) to control for potential biases from sampling *Hamiltonella* from aphid mummies. This may arise if there are limitations to symbiont detection in the DNA from aphid mummies or if selection from parasitoids biased the symbiont genotypes retained in mummies. For robustness, we analysed both the full data set of 1257 individuals and a conservative data set that only included the 1016 samples collected in 2021 and 2022 from mummified aphids. Analyses run on both data sets were qualitatively similar (Figures S4 and S5); however, the reduced sample size (number of pairwise comparisons between species) and increased predictor co‐linearity in the conservative data set reduced statistical power in some cases (e.g., Table S6) (Graham 2003).

Aphid‐parasitoid associations were identified using two methods: (1) Illumina amplicon sequencing of collected mummies, and (2) culturing live aphids until mummies formed and sequencing the emerged wasps. Mummified aphids were preserved in 70% ethanol for Illumina amplicon sequencing of the cytochrome oxidase subunit I (COI) gene to identify both parasitoid and aphid species. Live aphids, morphologically identified to species, were kept at 15°C with a 16 h:8 h light cycle for two weeks on host plant cuttings to harvest additional parasitoids. Emerging parasitoids were isolated, COI barcoded, and Sanger‐sequenced at Source Bioscience Ltd. (London, UK) for identification. Morphologically identified aphids were also confirmed by Sanger sequencing. We confirmed COI barcodes accurately identified parasitoids to species in five samples emerging from *Acyrthosiphon pisum* (Harris, 1776) and *Macrosiphum euphorbiae* (Thomas, 1878) by morphologically identifying emerged parasitoids (*Aphidius ervi* (Haliday, 1834) from *A. pisum* , and *Ephedrus californicus* (Baker, 1909), *Aphidius rhopalosiphi* (de Stefani‐Perez, 1902), from *M. euphorbiae* ) prior to sequencing. DNA extractions were performed using DNeasy Blood and Tissue Kits (QIAGEN, Venlo, Netherlands). DNA from mummies was normalised to 5 ng/μL. A 407 bp COI gene region was amplified and sequenced using CS1/CS2 tagged primers and a modified PCR protocol (Table S5). Tagged PCR products were sequenced at the London Genome Centre. We sequenced 1108 mummies in two Illumina MiSeq runs using paired‐end 300 bp reads: 370 samples in 2021 with 30,743 reads per sample and 738 samples in 2022 with 26,281 reads per sample. Sequencing data was analysed using the DADA2 pipeline (Callahan et al. 2016), with reads trimmed for quality, deduplicated, and chimeric sequences removed. Species were identified by comparing COI sequences to GenBank using BLASTn. Sequences were aligned using the MUSCLE aligner in MEGA11 (Tamura et al. 2021).

Out of 1108 sequenced mummies, 833 yielded COI data for both parasitoid and aphid, with 129 detecting *Hamiltonella* for multilocus sequence‐typing (MLST). An additional 183 parasitoid samples identified by Sanger sequencing brought the total to 1016 parasitoid‐aphid‐*Hamiltonella* samples. Transient associations, where single aphids were collected from unrecognised host plants, were removed from the data set. Non‐mummified samples and those where *Hamiltonella* was not detected were scored as having ‘No Association’ for parasitoid or symbiont and removed from corresponding analyses. Combined, these data sets resulted in 1257 samples for tripartite analyses.

### 2.2 *Hamiltonella* Strain Identification

Samples from 2021/2022 were screened for *Hamiltonella* using diagnostic PCR targeting the 16S rRNA gene (Niepoth et al. 2018) (Table S5). Positive samples were genotyped using MLST of four bacterial housekeeping genes: accD, hrpA, murE, and recJ (Degnan and Moran 2008) (Table S5). PCR products were Sanger‐sequenced in one direction and aligned using the MUSCLE aligner in MEGA11 (Tamura et al. 2021). *Hamiltonella* sequences from aphids collected between 2011 and 2019 (Wu et al. 2022) were included in the alignment. The four genes were concatenated for analyses.

### 2.3 Clustering of Operational Taxonomic Units

COI gene sequences exhibiting over 99% similarity were clustered into a single operational taxonomic unit (OTU). Species identifications for OTUs were based on the closest matching sequences in GenBank, with those > 99% similarity assigned morphospecies names (sp1, sp2, etc.). Multiple parasitoid OTUs matching the same species were designated as separate lineages (e.g., ‘clade 1’, ‘clade 2’) in analyses. For *Hamiltonella*, each MLST type was considered a separate OTU and referred to as a ‘strain’.

### 2.4 Phylogeny Reconstruction

Phylogenetic trees for aphids (COI), parasitoids (COI), and *Hamiltonella* (MLST) were constructed using PhyML (Guindon et al. 2010) with 100 bootstrap replications on the ATGC bioinformatics platform (http://www.atgc‐montpellier.fr/), visualised and rooted using iTOL software (Letunic and Bork 2024). *Adelges cooleyi* (Gillette, 1907), *Formica fusca* (Linnaeus, 1758), and *Hamiltonella* strain MEAM1 of *Bemisia tabaci* (Gennadius, 1889) were used as roots for aphid, parasitoid, and symbiont phylogenies, respectively. Bayesian Information Criterion (BIC) was used for model selection through Smart Model Selection (SMS) (Lefort et al. 2017).

### 2.5 Network Visualisations and Specialisation Tests

Network visualisations were conducted using R v4.2.2 (R Core Team 2023) in RStudio. The aphid‐*Hamiltonella*‐plant and aphid‐*Hamiltonella*‐parasitoid interactions (Figure 2) were mapped using the ‘igraph’ package (Csárdi et al. 2024) with the Fruchterman‐Reingold layout. Networks showed similar patterns when reduced to data collected in 2021 and 2022 (Figure S4). Bipartite plots (Figure 4) were constructed using the ‘plotweb’ command in the ‘bipartite’ package (Dormann et al. 2008), assigning *Hamiltonella* strains to parasitoids proportionally. The *Hamiltonella‐*aphid interaction matrix was plotted using the ‘ape’ package (Paradis and Schliep 2019) as in Wu et al. (2022). Network specialisation was tested using the H2′ index (Blüthgen et al. 2007) by comparing observed H2′ values with those from the null models using 1000 randomisations to generate standardised effect sizes.

### 2.6 Statistical Analysis

Statistical analyses were conducted in R v4.2.2. Relationships between *Hamiltonella* strain diversity and parasitoid or plant diversity were tested using a linear model in the ‘lme4’ package (Bates et al. 2015), visualised in ggplot2 (Wickham 2016) and ‘igraph’ (Csárdi et al. 2024). Data were rarefied (*n* = 1000 randomisations) to account for differences in sample sizes using the ‘rrarefy’ function in ‘vegan’ (Oksanen et al. 2022). Diversity indexes were calculated using custom scripts. To test which ecological variables are the best predictors of *Hamiltonella* genotypes shared by aphid species pairs, we generated Bray–Curtis dissimilarity matrixes for parasitoid, plant, and *Hamiltonella* communities using the ‘vegan’ package. For ease of interpretation in our network analyses (e.g., Figure 2), matrix values were converted to ‘similarity’ matrixes, using 1‐Bray Curtis, where 1 is most similar and 0 is no similarity. For consistency, we used similarity matrixes and the term “community similarity” throughout. To control for the potential influence of aphid phylogenetic relatedness on *Hamiltonella*'s distribution, we analysed our data using a multiple matrix regression with randomization (MMRR) test with aphid phylogenetic relatedness and parasitoid (or plant) community similarity as multiple independent predictor matrices using script adapted from (Wang 2013). The aphid phylogenetic relatedness matrix was based on our COI data and generated using the ‘dist.gene’ function in ‘ape’. Aphid species lacking data for either parasitoids or *Hamiltonella* strains were excluded from analyses to prevent missing data from impacting results. This ensured accurate interpretation of relationships amongst aphids, parasitoids, and *Hamiltonella* strains.

## 3 Results

### 3.1 Charactering the Parasitoid and Plant Associations of Aphids

**FIGURE 1:**
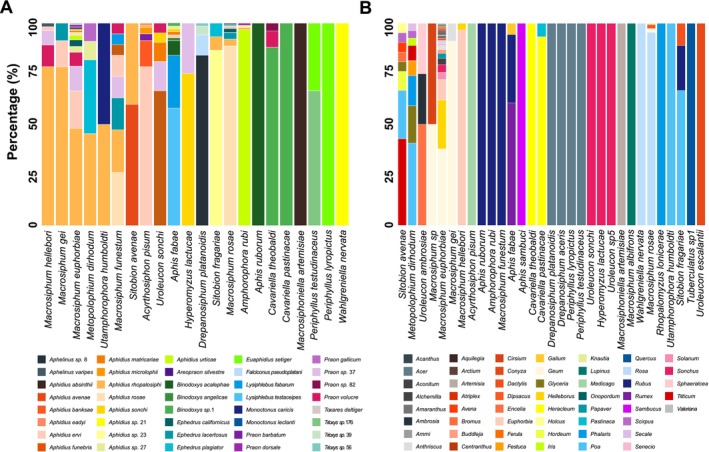
Relative frequency of parasitoid and host plant associations across aphids. Each bar represents an aphid species with different colours denoting the relative abundance of (A) different parasitoid species attacking the aphids and (B) different plant genera on which the aphids feed. Bars are organised according to the similarity of parasitoid/host plant communities as determined by hierarchical clustering. For visualisation purposes, different OTUs, or ‘clades’ belonging to a single parasitoid species, were merged into a single colour.

Our analysis of the 1257 samples identified 45 parasitoid species and 36 *Hamiltonella* strains associated with 31 aphid species collected from 62 species of plants (Figure 1, Tables S1 and S2). A phylogeny based on the parasitoid COI gene revealed that the parasitoid species belong to 40 species (5 sub‐species) from 12 genera and 2 Hymenopteran families, the Braconidae and Chalcididae. However, the vast majority (43/45) of aphid parasitoids were Braconids (Figure S1). Most parasitoid species (65%) were recovered from more than one aphid species. However, most parasitoids were primarily associated with a single aphid species or genus (although the host species varied between parasitoids) with only intermittent use of other aphid species (Figure 1). The majority (14/22 species, 64%) of aphid species were attacked by a single dominant parasitoid that accounted for > 75% of parasitism for each aphid species, and most aphid species (18/31 species, 58%) fed on a single dominant species of plant (although again, the dominant parasitoid and plant species varied between aphid species). There were also numerous cases where multiple aphid species shared the same host plant or parasitoid species (Figure 1, Table S2). This demonstrates that this is an appropriate system to test whether the sharing of plants or parasitoids is linked to the sharing of *Hamiltonella* strains across aphid species.

### 3.2 *Hamiltonella* Diversity Across Aphid Species

We were able to amplify *Hamiltonella* from 129 parasitoid mummies (Table S2). Genotyping the symbionts and placing them in a *Hamiltonella*‐aphid phylogenetic matrix previously generated by Wu et al. (2022), we identified 7 new *Hamiltonella* strains associated with this aphid community. We revealed that the newly identified strains tend to be associated with a single aphid species (Figure S2). For example, *Hamiltonella* strain N_1_88 was only found in *Periphyllus lyropictus* (Kessler, 1886) and was found at relatively high frequencies (47%, 16 out of 34) in this aphid. However, we also found new cases of the same symbiont strains being shared by distantly related host species. For example, the aphids *Cavariella theobaldi* (Gillette & Bragg, 1918) and *Macrosiphum funestrum* (Macchiati, 1885) were both found with *Hamiltonella* strain N_2_65 and the aphids *Aphis fabae* (Scopoli, 1763) and *M. funestrum* were both found harbouring strain N_4_75. In total, 16 out of 36 (44.4%) *Hamiltonella* strains were found across multiple aphid species. *Hamiltonella* strain 231 and strain 1485 are particularly noteworthy in their distributions; strain 231 was found in 15 aphid species across 7 genera and strain 1485 in six aphid species belonging to 3 genera. Our analysis also revealed several cases where a single aphid species was associated with two or more unrelated *Hamiltonella* symbiont strains. For example, *A. fabae* , *P. lyropictus*, *M. funestrum*, *Metopolophium dirhodum* (Walker, 1849), *Hyperomyzus lactucae* (Linnaeus, 1758), and the *Medicago* biotype of *A. pisum* were all associated with multiple unrelated *Hamiltonella* strains that they carried at relatively high frequencies (Figure S2).

We tested whether aphids associated with greater *Hamiltonella* diversity were associated with greater parasitoid or plant diversity using both rarefied and raw data sets (Table S3). Irrespective of the data set used, there was no correlation between *Hamiltonella* strain diversity, and the diversity of parasitoid or plant species associated with aphids (Figure S3, Table 1).

**TABLE 1:** General linear model (GLM) of the association between Hamiltonella strain diversity and parasitoid/host plant diversity in aphids.

Groups	Dataset	Richness			Shannon			Simpson		
		*R* ^2^	*F*	*p*	*R* ^2^	*F*	*p*	*R* ^2^	*F*	*p*
Parasitoid	Full dataset	0.025	1.538	0.229	0.072	2.640	0.120	0.044	1.962	0.177
Parasitoid	Rarefied *n* = 7	−0.037	0.537	0.478	−0.022	0.719	0.413	−0.026	0.673	0.428
Parasitoid	Mummies only	−0.068	0.041	0.842	0.002	1.029	0.328	0.009	1.135	0.305
Plant	Full dataset	0.012	1.368	0.252	0.025	1.767	0.194	0.007	1.210	0.280
Plant	Rarefied *n* = 7	−0.056	0.040	0.844	−0.058	0.007	0.932	−0.058	0.008	0.932
Plant	Mummies only	−0.043	0.382	0.546	−0.064	0.100	0.756	−0.065	0.092	0.767

*Note:* Three data set were tested, including the full data set, rarefied (*n* = 7) data set and our conservative data set based on mummies only.

### 3.3 Aphid‐*Hamiltonella* Associations Are Correlated With Parasitoid Attack Networks

**FIGURE 2:**
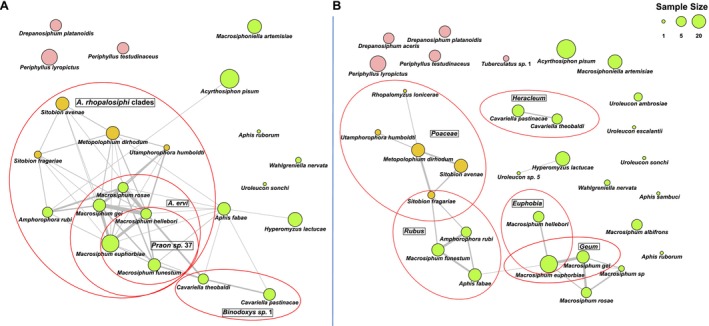
Parasitoid‐*Hamiltonella* and plant‐*Hamiltonella* network similarity of aphids. Grey lines connect aphid species that share similar *Hamiltonella* and (A) parasitoid species or (B) plant species. The thickness of each line corresponds to the sum of the similarity (1‐Bray Curtis distance) values of parasitoids/plants and *Hamiltonella* communities (Table S4). The size of each node reflects the number of *Hamiltonella* samples collected for each aphid species, while the colour denotes different aphid‐plant classification: Pink for tree‐dwelling aphids, yellow for predominantly grass‐dwelling aphids (although some host alternate), and green for herb‐dwelling aphids. Red circles highlight predominant parasitoid and host plant associations (at > 0.1 community similarity) that also share *Hamiltonella* strain(s). Parasitoid species and host plant genera (or families) are indicated in black squares.

**FIGURE 3:**
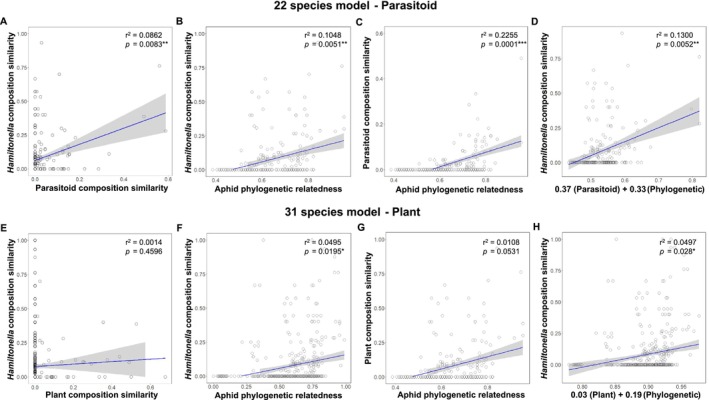
Modelling *Hamiltonella* genotype composition similarity in relation to parasitoid and plant community similarity and aphid phylogenetic relatedness. All panels visualise multiple matrix regression with randomization analysis (MMRR) using scatterplots. Scatterplots show the relationship of (A, E) parasitoid/plant community and *Hamiltonella* composition similarity, (B, F) aphid phylogenetic relatedness and *Hamiltonella* composition similarity, and (C, G) aphid phylogenetic relatedness and parasitoid/plant composition similarity. Panel (D) represents the inferred multiple regression effects of parasitoid (*β*_par_ = 0.37) and aphid phylogenetic relatedness (*β*_aph_ = 0.33) and (H) the inferred multiple regression effects of plant (*β*_pla_ = 0.03) and aphid phylogenetic relatedness (*β*_aph_ = 0.19) on *Hamiltonella* composition similarity. Panels (A–D) use the 22‐species model for parasitoid analysis, whereas panels (E–H) use the 31‐species model for plant analysis.

Comparing community similarity (1‐Bray Curtis) indexes, we found that aphid species that shared the same parasitoid species tended to harbour the same *Hamiltonella* strains (Figure 2A). However, aphid pairs that fed on the same species of plants had fewer incidences of sharing *Hamiltonella* strains (Figure 2B). To test the relative importance of ecological variables on *Hamiltonella* genotype distributions, we used an MMRR test with multiple predictor matrices that included host aphid phylogenetic relatedness and parasitoid (or plant) community composition. Both parasitoid community similarity and aphid phylogenetic relatedness were important in explaining *Hamiltonella* community similarity when both predictors were included in a single model (MMRR test: *β*_*par*_ = 0.3670, *p* = 0.0465*; *β*_*Aph*_ = 0.3253, *p* = 0.0748; Table S6). Aphid species with similar *Hamiltonella* communities shared more similar parasitoid communities, and these aphid species also tended to be more phylogenetically closely related (Figure 3). In contrast, analysing the plant‐*Hamiltonella* data with aphid relatedness as a predictor demonstrated plants had no influence on *Hamiltonella*'s distribution (MMRR test: *β*_*Pla*_ = 0.0344, *p* = 0.7841), but aphid genetic relatedness did (MMRR test: *β*_*Aph*_ = 0.1972, *p* = 0.0217*) (Figure 3; Table S6). However, most aphid species in our data set were collected from a single plant species, which may have limited our capacity to detect some plant‐based effects.

Within the parasitoid‐aphid‐*Hamiltonella* data, we identified 85 pairs of aphid species that shared *Hamiltonella* strains; amongst these, 47 pairs (55.3% of pairwise comparisons) exhibited varying degrees of similarity in their parasitoid compositions (Table S4). 15 out of 19 (78.9%) aphid species sharing *Hamiltonella* strains could be linked by sharing at least one parasitoid species (Table S4). This is particularly evident in the *Macrosiphum* genus, which has the highest similarity in parasitoid species that attack them and tend to harbour the same *Hamiltonella* strains (average parasitoid community similarity: 0.244, average *Hamiltonella* community similarity: 0.423, Table S4: where 1 indicates identical communities and 0 is no similarity (i.e., 1‐ Bray Curtis distance)). Several aphids that feed on grasses, including *Utamphorophora humboldti* (Essig, 1941), *M. dirhodum*, *Sitobion avenae* (Fabricius, 1775), and *Sitobion fragariae* (Walker, 1848), showed a relatively high degree of parasitoids (0.156) and *Hamiltonella* community similarity (0.074) with each other, as well as with *Macrosiphum* aphids (average parasitoid and *Hamiltonella* community similarity = 0.041 and 0.131, respectively, Table S4). The two *Caveriella* species, *C. theobaldi* and *C. pastinacae* (Linnaeus, 1758), are also predominately attacked by the same parasitoid (Parasitoid community similarity = 0.33) and have evidence of sharing the same *Hamiltonella* strain 231 (*Hamiltonella* community similarity = 0.11), but also feed on the same host plant, *Heracleum sphondylium* (Linnaeus, 1753).

The plant‐aphid‐*Hamiltonella* data revealed 138 pairs of aphid species that shared symbiont strains; yet only 20 pairs (14.5% of pairwise comparisons) had similarities in host plant species (Figure 2B, Table S4). Furthermore, 8 aphid species, *A. pisum, Aphis ruborum* (Börner, 1931), *Aphis sambuci* (Linnaeus, 1758), *Macrosiphum albifrons* (Essig, 1911), *P. lyropictus, Uroleucon escalantii* (Knowlton, 1928), *Uroleucon sonchi* (Linnaeus, 1767), and *Wahlgreniella nervata* (Gillette, 1908), shared *Hamiltonella* strains with other aphid species but were not found sharing any host plants in our data set. Unlike the parasitoid‐aphid‐*Hamiltonella* interactions that linked all correlated aphid species together, the plant‐aphid‐*Hamiltonella* interaction formed three groups: (i) 3 of 4 aphids feeding on *Rubus* are grouped with aphids that feed on grasses (Poaceae) through the host‐plant alternating *Sitobion fragariae*, which are then weakly connected to 3 *Macrosiphum* species feeding on *Geum*; (ii) 2 of 3 species on *Sonchus* share some similarity; and (iii) the two *Cavariella* species that feed on *Heracleum* share plants, parasitoids, and *Hamiltonella* strains (Figure 2).

Notably, there were 4 aphid species, *P. lyropictus*, *U. sonchi* , *W. nervata,* and *A. ruborum*, that shared *Hamiltonella* strains but did not share the same parasitoids or host plants. This indicates that there were still connections in the symbionts' distribution that cannot be explained by the plants or parasitoids identified in our data set.

### 3.4 Specialised Parasitoid‐Aphid‐*Hamiltonella* Associations

**FIGURE 4:**
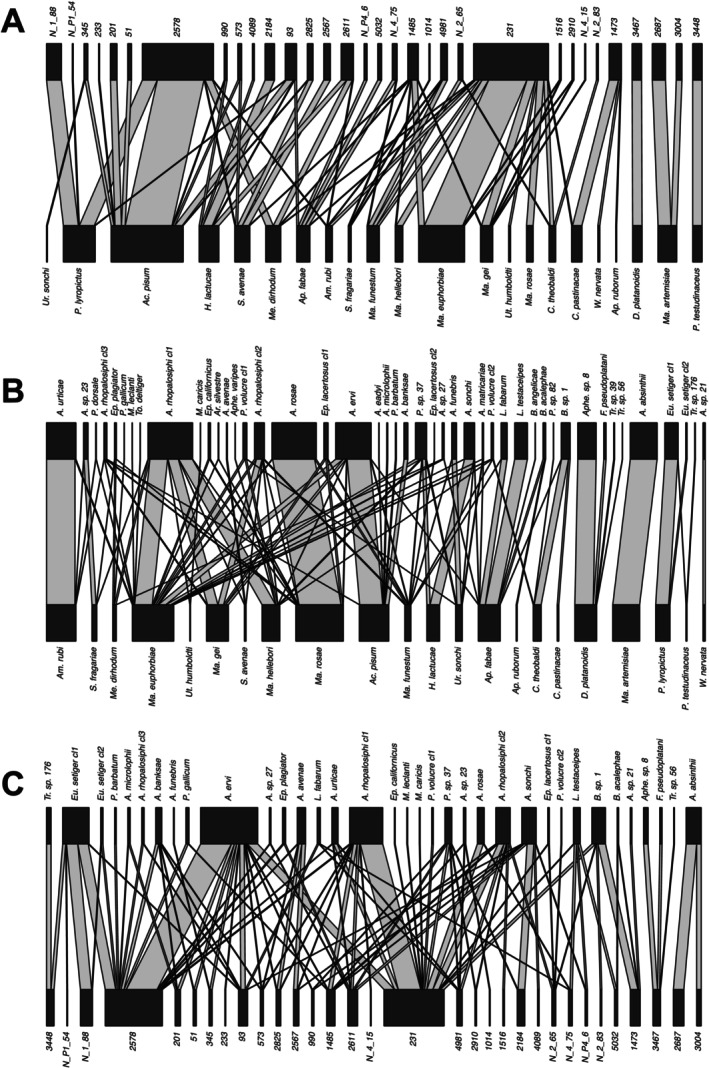
Bipartite parasitoid, aphid, and *Hamiltonella* interaction networks. Networks depict (A) *Hamiltonella* strain (top) – aphid species (bottom), (B) parasitoid species (top) – aphid species (bottom), and (C) parasitoid species (top) – *Hamiltonella* strain (bottom) interactions. The parasitoid‐*Hamiltonella* network was generated by assigning *Hamiltonella* “individuals” in each aphid species to parasitoids attacking them, proportional to the frequency of attacks across different parasitoid species. Grey lines connect interacting species and strains, and the width of the line indicates the frequency of interaction between pairs. The width of the black bars denotes the relative abundance of individual aphids, parasitoids, and *Hamiltonella* strains used in each bipartite network because these were assessed using different sets of samples.

Our analyses revealed high degrees of specialisation in all interaction networks, indicating aphids, parasitoids, and symbiont strains tend to interact with a limited number of partners (Figure 4). Specialisation (as measured using H2′) was highest for parasitoid‐aphid networks (standardised effect size (SES) = 153.3), but also high for both *Hamiltonella*‐aphid (SES = 48.3) and the generated *Hamiltonella*‐parasitoid (SES = 34.0) networks. Most aphid species harboured a single dominant *Hamiltonella* strain and were primarily targeted by a specific parasitoid species (Figure 4A,B). For example, in the *Macrosiphum* genus, particularly *Macrosiphum gei* (Koch, 1855), *M. euphorbiae* , and *Macrosiphum hellebori* (Theobald & Walton, 1923), all species predominantly harboured *Hamiltonella* strain 231 and were mainly attacked by a single parasitoid species, *Aphidius rhopalosiphi* clade 1. Notably, *A. rhopalosiphi* clade 1 was the most generalist parasitoid in our study, attacking 8 aphid species belonging to 4 genera; 6 of the species carried *Hamiltonella* strain 231. In addition to *A. rhopalosiphi* clade 1, *Macrosiphum* aphids were attacked by a similar group of parasitoids at lower incidences, including *A. rhopalosiphi* clades 2 and 3, and several species from the *Aphidius*, *Praon*, and *Ephedrus* genera (Figure 4B). *Aphis fabae* also maintained diverse connections in that it shared *Hamiltonella* strains and parasitoids attacking it at relatively low frequencies with 10 other aphid species from 4 genera, including *Acyrthosiphon*, *Cavariella*, *Macrosiphum*, *Metopolophium*, and *Hyperomyzus* (Figure 4, Tables S1 and S4). Conversely, there were also aphid species, such as *Drepanosiphum platanoidis* (Schrank, 1801) and *Macrosiphoniella artemisiae* (Boyer de Fonscolombe, 1841), that harboured a unique cluster of *Hamiltonella* strains and were each attacked either primarily or exclusively by a single parasitoid species. The parasitoid‐*Hamiltonella* network also revealed several cases where parasitoids had particularly strong associations with certain symbiont strains, such as *A. rhopalosiphi* and *A. ervi*, which are linked to *Hamiltonella* strains 231 and 2578, respectively.

## 4 Discussion

We demonstrate that natural enemy attack networks are linked to the distribution of defensive symbiont strains within an insect community. Population surveys, including ours, show that facultative symbionts providing protection tend to be non‐randomly distributed across aphid species and plant‐adapted populations; only certain species and populations harbour them, often with few symbiont strains at high frequency (Henry et al. 2013, 2015; Wu et al. 2022). We show aphids are typically attacked by a single dominant parasitoid species, and after accounting for the influence of host species relatedness (Wu et al. 2022; McLean et al. 2019; Łukasik et al. 2015), aphid species sharing the same parasitoids, rather than food plants, tend to carry the same strains of *Hamiltonella*. This suggests that interactions with parasitoids and their hosts play a key role in the spread and maintenance of this defensive symbiosis within aphid communities.

### 4.1 Parasitoids as Selective Agents Shaping *Hamiltonella*'s Distributions

Parasitoids impose strong selective pressure for the evolution of resistance in aphids. We show each aphid species is frequently attacked by a single dominant parasitoid, demonstrating a high degree of host specialisation. We suggest parasitoid host specificity may shape *Hamiltonella* distributions across aphid species. Laboratory studies have shown *Hamiltonella* strains vary in their degree of protection and specificity against different parasitoid species (Cayetano et al. 2015; Martinez et al. 2016). For example, McLean and Godfray (2015) found *Hamiltonella* strains associated with pea aphids on *Lotus* plants protect against *Aphelinus abdominalis* (Dalman, 1820), but not *Aphidius ervi*, while strains on *Medicago* plants show the opposite trend. Frequent attacks by a single parasitoid species likely select for aphids carrying a single or few closely related *Hamiltonella* strains, as these provide strong protection against their main natural enemy. Laboratory studies support this, showing attack by a single parasitoid species can lead to aphids carrying a single highly protective symbiont strain (Hafer‐Hahmann and Vorburger 2020). Moreover, studies have found aphid species often carry *Hamiltonella* strains in nature that confer strong protection against their most common parasitoid (Gimmi and Vorburger 2021; Wu et al. 2022). We suggest parasitoid pressures likely result in the initial establishment of *Hamiltonella*‐aphid relationships, which then become co‐adapted due to repeated interactions, leading to hosts adapted to carry certain symbiont strains (Wu et al. 2022; McLean et al. 2019; Łukasik et al. 2015). Strikingly, we reveal that aphid species attacked by the same parasitoids tend to carry the same *Hamiltonella* strains (based on 4 MLST genes), although in some cases the aphids are also related which may contribute to the association. This suggests aphids retain similar symbiont strains to protect against a shared enemy. While the molecular mechanism of protection is not fully resolved, the genomes of most *Hamiltonella* contain a toxin‐encoding bacteriophage known as APSE that is likely involved (Lynn‐Bell et al. 2019). Variation in APSE's toxin genes is thought to determine the degree of protection against the parasitoid *Aphidius ervi* (Oliver and Higashi 2019). It would be interesting to know whether APSE toxin variability also explains protection against different parasitoid species.

Although parasitoid and *Hamiltonella* community compositions were similar, there was no correlation between parasitoid species diversity and *Hamiltonella* strain diversity in aphids. Increased parasitoid diversity may not lead to increased symbiont diversity if there is a cost to resistance (Hafer‐Hahmann and Vorburger 2024). Furthermore, factors such as transmission efficiency, host‐symbiont compatibility, or interactions with other microbes, pathogens, or the environment may also impact *Hamiltonella* diversity (Carpenter et al. 2021; Dykstra et al. 2014; Niepoth et al. 2018; Weldon et al. 2020; Goldstein et al. 2023). We also did not consider genotype diversity within parasitoid species, which may influence symbiont diversity associated with an aphid species (Hafer‐Hahmann and Vorburger 2020).

### 4.2 Influence of Plants on *Hamiltonella* Distribution

Studies show facultative symbionts occur more frequently in insect populations on certain plants, suggesting plant interactions may shape their distributions (Ferrari et al. 2004; Henry et al. 2013, 2015; Toju and Fukatsu 2011; Tsuchida et al. 2002). Examples include pea aphid biotypes on *Medicago sativa* (Linnaeus, 1753), *Lotus pedunculatus* (Cavanilles, 1793), and *Ononis* plants carrying biotype‐specific *Hamiltonella* strains, and *A. fabae* populations feeding on different plants differing in *Hamiltonella* carriage (Gimmi et al. 2024; Henry et al. 2013, 2015). However, our results indicate there is no link between host plant sharing and the sharing of *Hamiltonella* strains in aphids. This suggests that plants have a limited role in shaping *Hamiltonella* distribution, at least amongst aphid species. Aphids on different plants might attract different parasitoid species due to changes in plant volatile profiles, or *Hamiltonella* itself may modify plant volatiles to attract different parasitoid species (Ahmed et al. 2022; Ali et al. 2022; Frago et al. 2017), leading to plant‐associated *Hamiltonella* strains. It would be interesting to determine if plant‐adapted aphid biotypes are attacked by different parasitoid species, as this may explain their tendency to carry different *Hamiltonella* strains.

### 4.3 Ecological Vectors of *Hamiltonella* Transmission

Hypotheses explaining horizontal transmission of facultative symbionts amongst insects include natural enemy and plant sap transmission. Natural enemies might pick up and transmit symbionts via contaminated mouthparts or ovipositors (Ahmed et al. 2015; Gehrer and Vorburger 2012; Kaech and Vorburger 2021; Soleimannejad et al. 2023; Tzuri et al. 2021), while plant sap transmission involves symbionts being released into plant sap and acquired by another insect ingesting it (Chrostek et al. 2017; Li et al. 2018; Pons et al. 2019). We show that the same *Hamiltonella* strains occur in distantly related aphids attacked by the same parasitoid species. Moreover, aphids carrying low frequencies of *Hamiltonella* strains typically found in other aphids are often also attacked by their parasitoids at low incidences. This suggests parasitoids may be a vector of symbiont transmission in wild aphid populations. Laboratory studies have shown *Hamiltonella* can be transmitted by parasitoids in *A. fabae* , with transmission success depending on symbiont titre and haplotype (Kaech and Vorburger 2021). The likelihood of symbiont transfer can also be influenced by the relatedness of the hosts and the symbionts they previously harboured (Łukasik et al. 2015; McLean et al. 2019). Parasitoids have also been shown to transfer facultative symbionts in other insects, such as *Myzus persicae* (Sulzer, 1776) and *Bemisia tabaci* , and in house flies, *Musca domestica* (Linnaeus, 1758) (Ahmed et al. 2015; Soleimannejad et al. 2023; Tzuri et al. 2021). In contrast, we find little evidence that sharing the same host plants leads to sharing *Hamiltonella* strains. Plant‐mediated horizontal transfer has been reported in several sap‐feeding insects under laboratory conditions (Caspi‐Fluger et al. 2012; Gonella et al. 2015; Li et al. 2017), including *Hamiltonella*, where plant transfer has been shown in *Sitobion miscanthi* (Takahashi, 1921) feeding on wheat (Li et al. 2018). We find that when aphid species do share the same food plant and *Hamiltonella* strains, they also share the same parasitoids. Moreover, there are no cases where plant sharing alone explains *Hamiltonella*'s distribution, even in aphids that have a strong similarity in plant use, and do not share parasitoids. This suggests horizontal transfer by plants is either infrequent in aphids or potentially only occurs in certain plants. It is also possible that selection from parasitoids may rapidly purge plant‐transferred symbiont strains from aphid populations, thereby contributing to the observed pattern. However, in most cases where *Hamiltonella* strains are shared between aphid species, it cannot be explained by plants, suggesting if it does occur, it is of limited importance.

### 4.4 Facultative Symbionts as a Reservoir of Adaptations in Insect Defence

The non‐random distribution of facultative symbionts in insects has puzzled scientists, especially in bacteria known to provide hosts with protection. Our results suggest that selection from or lateral transfer by natural enemies alongside host relatedness are key factors shaping the distribution of defensive symbionts in aphids and potentially other insects. Specifically, the link between *Hamiltonella* strains and parasitoid networks supports the idea that coevolutionary dynamics have led to host specialisation in natural enemies that has been largely mediated by the symbiont (Vorburger 2022). This may be due to symbiont‐conferred defences being more specific than host‐encoded defences, allowing hosts to tailor defences towards specific enemies through symbiont acquisitions. Specificity in symbiont‐mediated protection has been reported in several defensive symbioses (Higashi et al. 2023; Łukasik et al. 2013; Mateos et al. 2016) and is particularly evident in *Hamiltonella* (McLean and Godfray 2015; Rouchet and Vorburger 2012; Wu et al. 2022). Bacteria have the potential to evolve more rapidly than the host's genome, particularly when genes involved in protection are located on mobile genetic elements, such as the diverse toxin genes contained on *Hamiltonella*'s APSE phage. Parasitoids can become resistant to the presence of defensive symbionts (e.g., Dion et al. 2011; Oliver et al. 2008). The arsenal of defences carried by *Hamiltonella*'s APSE phages, in combination with their potential to mobilise, may be the crucial component in winning the evolutionary arms race against parasitoids, leading to the widespread distribution of the symbiont in aphids. However, the selection from *Hamiltonella* on specific natural enemies may also disrupt host specialisation, causing parasitoids to switch hosts to avoid highly protective symbiont strains or providing an advantage to secondary parasitoids that attack a host at lower frequencies (e.g., McLean and Godfray 2017).

It has been suggested that horizontal transfer of defensive symbionts is rare enough that different host species carry distinct symbiont communities (Vorburger 2022). Our results suggest otherwise, as aphids attacked by the same parasitoid species tend to harbour related *Hamiltonella* strains. This suggests a more dynamic relationship where symbionts are maintained in host populations, at least across generations, at time scales that are relevant to selection from parasitoids. However, a finer‐scale genetic analysis of shared symbionts and phages is needed to confirm this. Nonetheless, horizontal transfer of symbionts by parasitoids, even if rare, likely contributes to the genetic similarity of *Hamiltonella* strains occurring in aphids that share the same parasitoids and provides an important source of incoming symbiont variants for selection to act upon. Future studies are needed to assess whether symbiont transfer is rapid enough to counteract changes in parasitoid communities or hamper parasitoid counteradaptations to determine exactly how insects use symbionts in their defence against natural enemies.

## Author Contributions

L.M.H. conceived the idea, acquired funding, and led the investigation and supervision of the team. T.W. and A.A.R. conducted field collections and formal analysis of the data. T.M.F. conducted additional analyses. All authors contributed to the drafting, reviewing, and editing of the manuscript. All authors gave final approval for publication and agreed to be held accountable for the work performed therein.

## Conflicts of Interest

The authors declare no conflicts of interest.

### Peer Review

The peer review history for this article is available at https://www.webofscience.com/api/gateway/wos/peer‐review/10.1111/ele.70082.

## Supporting information


**Figure S1.** Maximum likelihood phylogeny of parasitoid species associated with aphids based on the COI gene (Second segment, Ill_B_F/HCO2198).
**Figure S2**. Phylogenetic relationship of *Hamiltonella* strains identified in this study (light blue) in relation to previously known strains (royal blue) from Wu et al. (2022).
**Figure S3**. Correlations between *Hamiltonella* ecological indexes and (A) parasitoid ecological indexes or (B) host plant ecological indexes.
**Figure S4**. Parasitoid‐*Hamiltonella* (A) and plant‐*Hamiltonella* (B) network similarity using only mummy sample data collected in 2021 and 2022.
**Figure S5**. Modelling *Hamiltonella* genotype composition similarity in relation to parasitoid and plant community similarity and aphid phylogenetic relatedness using only mummy data collected in 2021 and 2022.
**Figure S6**. Null modelling of network specialisation.

Table S1.

## Data Availability

Data and scripts are available online: https://doi.org/10.5281/zenodo.12799210. Sequencing data is deposited in GenBank Bioproject PRJNA1139364. The GenBank accession numbers for Sanger‐sequenced *Hamiltonella* (MLST) are PQ475080 to PQ475552, and the accession numbers for Illumina‐sequenced aphids (COI) and parasitoids (COI) are PQ386310 to PQ386336 and PQ411044 to PQ411123, respectively.
