# Supplementary material for: Defensive Symbiont Genotype Distributions Are Linked to Parasitoid Attack Networks
Source: Ecol Lett. 2025 Feb 18;28(2):e70082. doi: 10.1111/ele.70082 (PMC11834374; doi:10.1111/ele.70082)
Supplement: Supplementary file 1 — Figure S1. Maximum likelihood phylogeny of parasitoid species associated with aphids based on the COI gene (Second segment, Ill_B_F/HCO2198). Figure S2. Phylogenetic relationship of Hamiltonella strains identified in this study (light blue) in relation to previously known strains (royal blue) from Wu et al. (2022). Figure S3. Correlations between Hamiltonella ecological indexes and (A) parasitoid ecological indexes or (B) host plant ecological indexes. Figure S4. Parasitoid‐Hamiltonella (A) and plant‐Hamiltonella (B) network similarity using only mummy sample data collected in 2021 and 2022. Figure S5. Modelling Hamiltonella genotype composition similarity in relation to parasitoid and plant community similarity and aphid phylogenetic relatedness using only mummy data collected in 2021 and 2022. Figure S6. Null modelling of network specialisation. [file ELE-28-0-s002.pdf]

SUPPLEMENTARY FIGURES

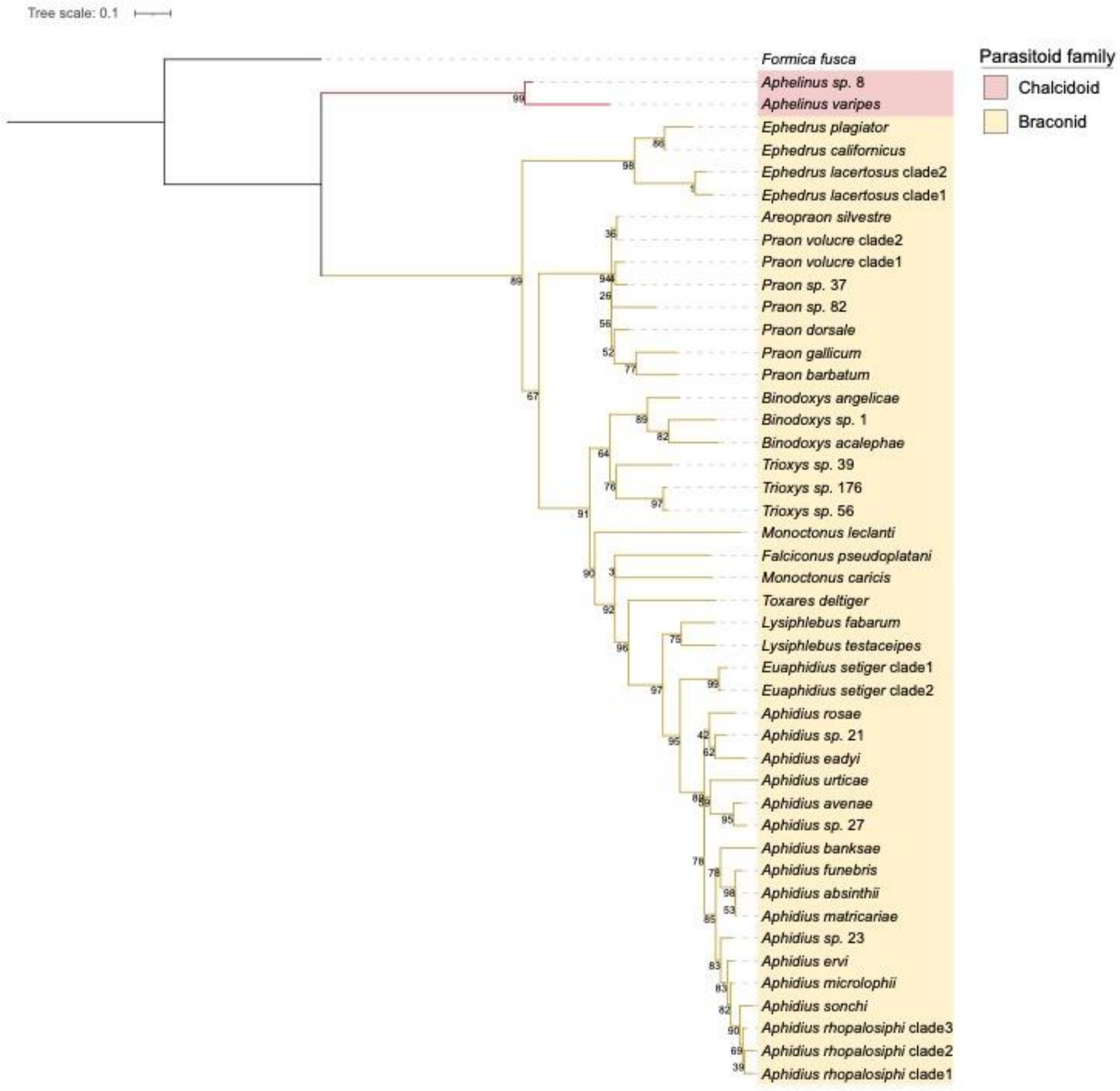

Figure S1: Maximum likelihood phylogeny of parasitoid species associated with aphids based on the COI gene (Second segment, Ill_B_F/HCO2198). Numbers at node represent support based on bootstrap values. Species are color-coded to represent parasitoid families: Yellow for Braconidae (Bracoid) and Red for Chalcididae (Chalcidoid).

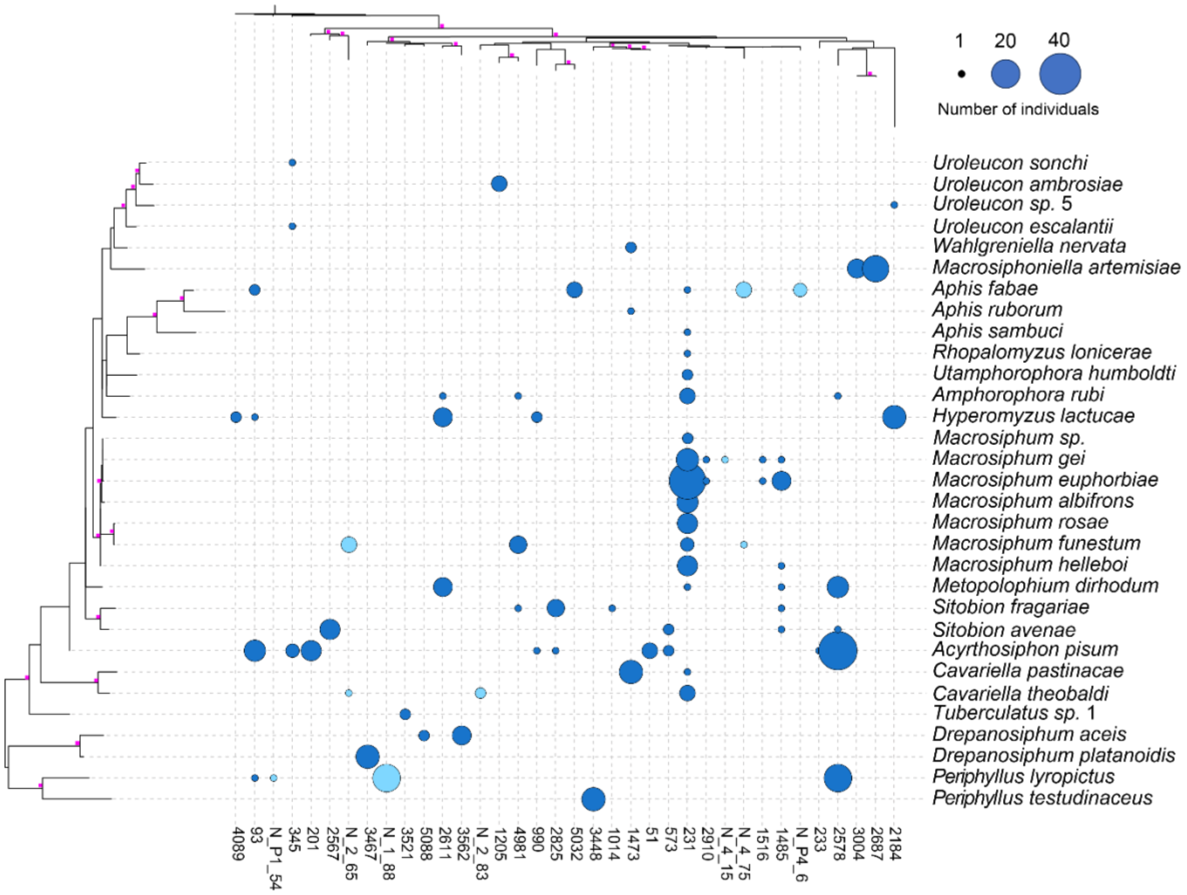

Figure S2. Phylogenetic relationship of *Hamiltonella* strains identified in this study (light blue) in relation to previously known strains (royal blue) from Wu *et al.* 2022. Interaction matrix displays *Hamiltonella* genotypes, based on 4 MLST genes (top phylogeny), occurring in aphid species, based on COI (left phylogeny). Squares on the phylogeny nodes denote branch support greater than 50. Bubble size corresponds to the number of times an aphid species was found harbouring a particular *Hamiltonella* genotypes.

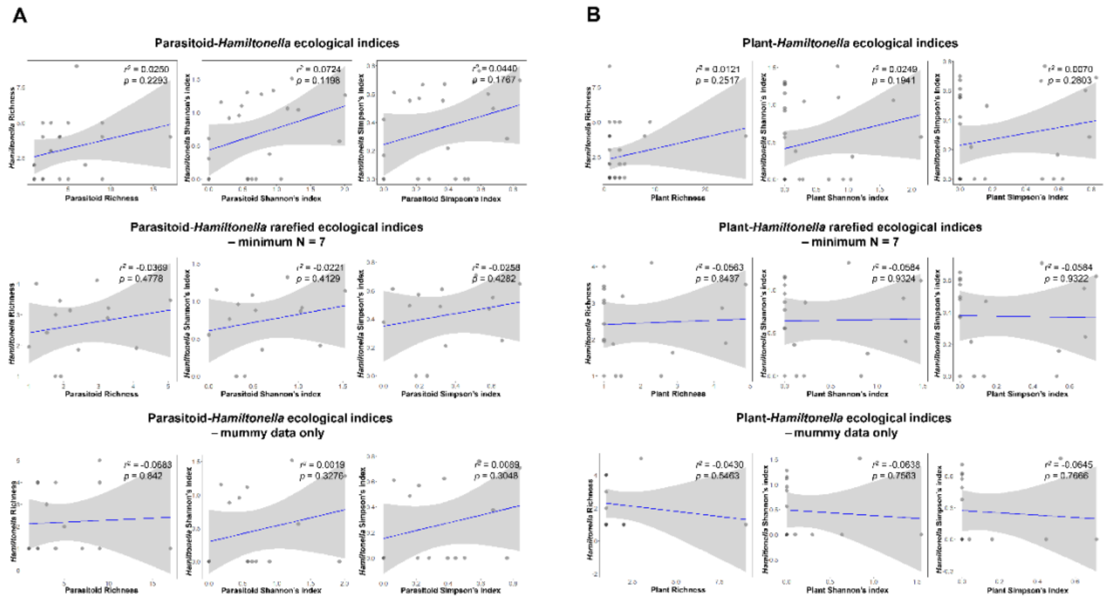

Figure S3: Correlations between *Hamiltonella* ecological indexes and A) parasitoid ecological indexes or B) host plant ecological indexes. The full data set is displayed in the top panel, the rarefied data set (rarefied to n = 7) is in the middle panel, and the conservative, mummy-only data is at the bottom. Within each column, three ecological indexes were compared, richness (left), Shannon's index (middle) and Simpson's index (right). Parasitoid/plant indexes are plotted on the x-axis, while *Hamiltonella* indexes are plotted on the y-axis. Adjusted R-squared values and p-values of the general linear model, as well as the regression line and 95% CI was shown in the top right of each panel.

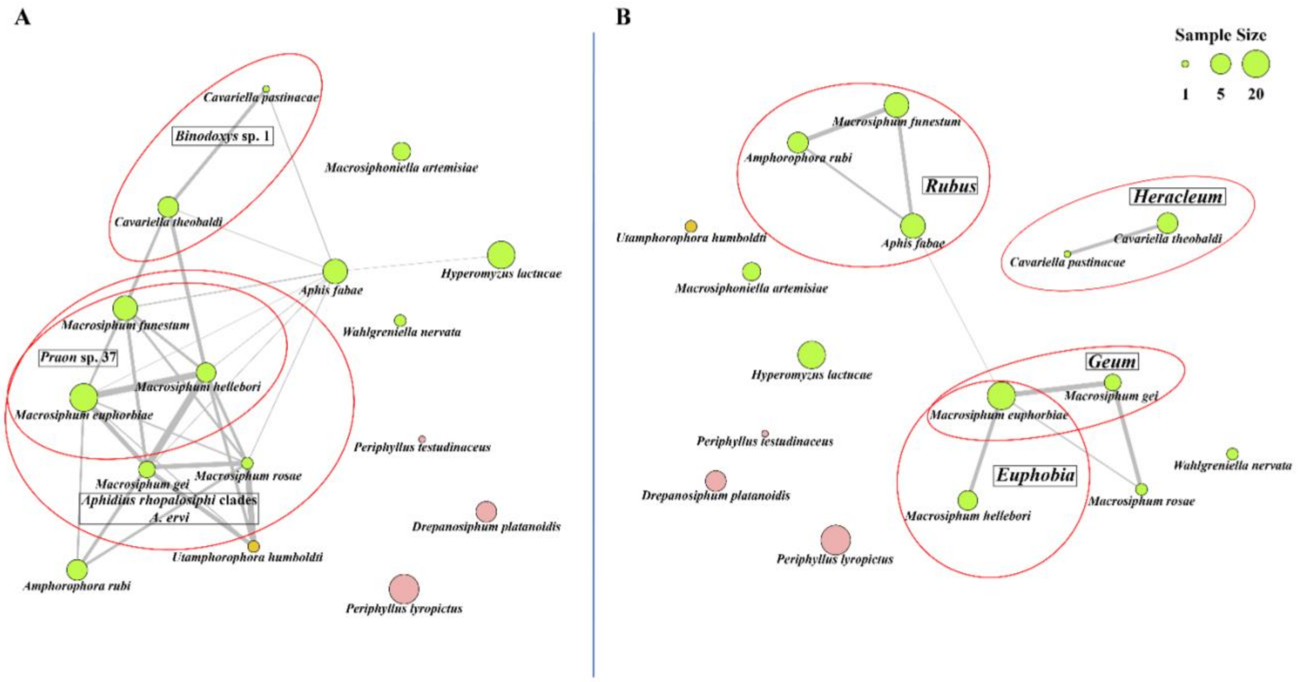

Figure S4: Parasitoid-*Hamiltonella* (A) and plant-*Hamiltonella* (B) network similarity using only mummy sample data collected in 2021 and 2022. Grey lines connect aphid species that share similar *Hamiltonella* and (A) parasitoid or (B) plant diversity. The thickness of each line corresponds to the sum of the Bray-Curtis similarity values of parasitoids/plant and *Hamiltonella* communities (Table S4). The size of each node reflects the number of *Hamiltonella* samples collected for that aphid species, while the colour denotes different aphid-plant classification: pink for tree-dwelling aphids, yellow for predominantly grass-dwelling aphids, and green for herb-dwelling aphids. Red circles highlighted the aphid species that share *Hamiltonella* strain(s) and also have common parasitoid or host plants. Shared parasitoid species and host plant genus are shown in black squares.

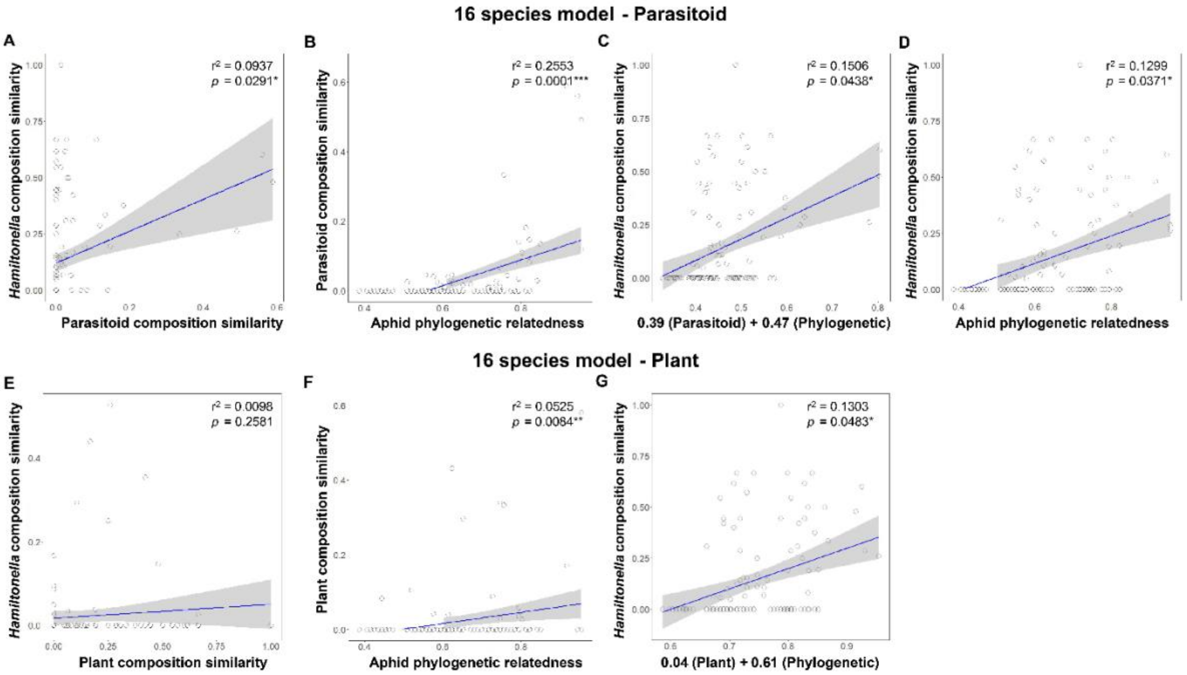

Figure S5: Modelling *Hamiltonella* genotype composition similarity in relation to parasitoid and plant community similarity and aphid phylogenetic relatedness using only mummy data collected in 2021 and 2022. All panels visualise Multiple matrix regression with randomization analysis (MMRR) using scatterplots. Scatterplots show the relationship of (A, E) parasitoid/plant community and *Hamiltonella* composition similarity, (B, F) aphid phylogenetic relatedness and parasitoid/plant composition similarity. Panel (C) represents the inferred multiple regression effects of parasitoid (β_{par} = 0.39) and aphid phylogenetic relatedness (β_{aph} = 0.47) and (G) the inferred multiple regression effects of plant (β_{pla}= 0.04) and aphid phylogenetic relatedness (β_{aph} = 0.61) on *Hamiltonella* composition similarity. Panel D is the aphid phylogenetic relatedness and *Hamiltonella* composition similarity for both models (plants and parasitoids).

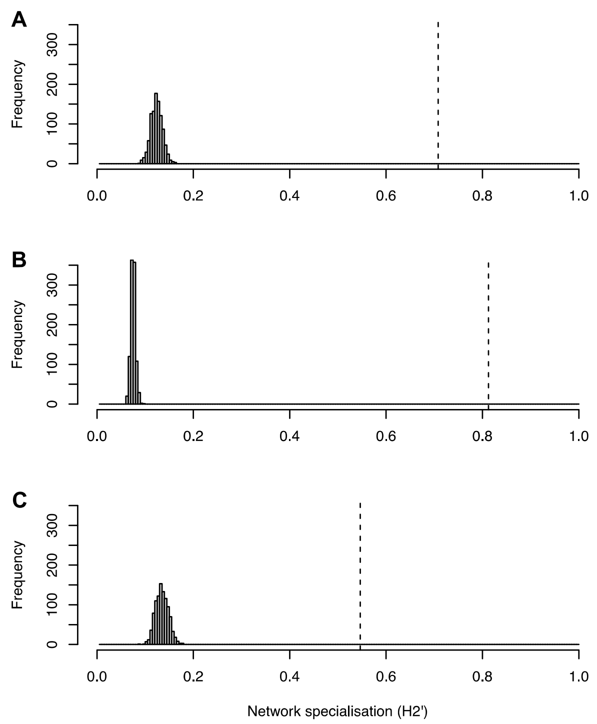

Figure S6: Null modelling of network specialisation. Network specialization distributions for (A) *Hamiltonella*-aphid, (B) parasitoid-aphid, and (C) *Hamiltonella*-parasitoid networks. Histograms show H2’ from null models, vertical broken lines show observed values of H2’.
